# Effect of tualang honey against KA-induced oxidative stress and neurodegeneration in the cortex of rats

**DOI:** 10.1186/s12906-016-1534-x

**Published:** 2017-01-09

**Authors:** Nur Shafika Mohd Sairazi, Sirajudeen K.N.S., Mohd Asnizam Asari, Swamy Mummedy, Mustapha Muzaimi, Siti Amrah Sulaiman

**Affiliations:** 1 Department of Chemical Pathology, School of Medical Sciences, Universiti Sains Malaysia, Health Campus, 16150 Kubang Kerian, Kota Bharu, Kelantan Malaysia; 2 Department of Anatomy, School of Medical Sciences, Universiti Sains Malaysia, Health Campus, 16150 Kubang Kerian, Kota Bharu, Kelantan Malaysia; 3 Department of Neurosciences, School of Medical Sciences, Universiti Sains Malaysia, Health Campus, 16150 Kubang Kerian, Kota Bharu, Kelantan Malaysia; 4 Department of Pharmacology, School of Medical Sciences, Universiti Sains Malaysia, Health Campus, 16150 Kubang Kerian, Kota Bharu, Kelantan Malaysia

**Keywords:** Tualang honey, Kainic acid, Neurodegeneration, Oxidative stress, Behavioral change, Fluoro Jade C, Cresyl violet

## Abstract

**Background:**

Administration of KA on rodents has resulted in seizures, behavioral changes, oxidative stress, and neuronal degeneration on selective population of neurons in the brain. The present study was undertaken to investigate the extent of neuroprotective effect conferred by Malaysian Tualang Honey (TH), an antioxidant agent, in the cerebral cortex of rats against KA-induced oxidative stress and neurodegeneration in an animal model of KA-induced excitotoxicity.

**Methods:**

Male Sprague–Dawley rats were randomly divided into five groups: Control, KA-treated group, TH + KA-treated group, aspirin (ASP; anti-inflammatory agent) + KA-treated group and topiramate (TPM; antiepileptic agent) + KA-treated group. The animals were pretreated orally with drinking water, TH (1.0g/kg BW), ASP (7.5mg/kg BW) or TPM (40mg/kg BW), respectively, five times at 12 h intervals. KA (15mg/kg BW) was injected subcutaneously 30 min after last treatment to all groups except the control group (normal saline). Behavioral change was observed using an open field test (OFT) to assess the locomotor activity of the animals. Animals were sacrificed after 2 h, 24 h and 48 h of KA administration.

**Results:**

KA significantly inflicted more neuronal degeneration in the piriform cortex and heightened the predilection to seizures as compared with the control animals. Pretreatment with TH reduced the KA-induced neuronal degeneration in the piriform cortex but failed to prevent the occurrence of KA-induced seizures. In the OFT, KA-induced animals showed an increased in locomotor activity and hyperactivity and these were attenuated by TH pretreatment. Furthermore, TH pretreatment significantly attenuated an increase of thiobarbituric acid reactive substances level and a decrease of total antioxidant status level enhanced by KA in the cerebral cortex.

**Conclusion:**

These results suggest that pretreatment with TH has a therapeutic potential against KA-induced oxidative stress and neurodegeneration through its antioxidant effect.

**Electronic supplementary material:**

The online version of this article (doi:10.1186/s12906-016-1534-x) contains supplementary material, which is available to authorized users.

## Background

Excitotoxicity has been considered to be a major pathological process of neuronal death in acute and chronic neurodegenerative diseases such as Alzheimer’s disease, Parkinson’s disease, Huntington’s disease, temporal lobe epilepsy (TLE), amyotrophic lateral sclerosis and hypoxia/ischemia [1]. Kainic acid (KA), a neurotoxicant extracted from red alga, Digenea simplex, is widely used to induce seizures and to explore the mechanism of excitotoxicity and neurodegeneration. Studies on KA-induced animal experimental model have shown series of clinical manifestations and pathological changes upon KA administration on rodents such as seizures [2], behavioral changes of rodents [3, 4], oxidative stress [5, 6] and neuropathological lesions akin to those found in patients with TLE [7].

In recent decades, there is an emerging trend to search for natural resources to combat against neurodegenerative diseases. Many studies have tested or reported the protective effect of various types of natural products against KA-induced excitotoxicity models [3, 8–11]. Many of natural products possess antioxidant activity that enables them to protect against KA –induced neuronal degeneration and KA-induced seizures. In Malaysia, Tualang honey (TH) is widely used as a nutrient supplementation and in traditional medicine. TH is a wild multiflora honey produced by Asian rock bees (Apis Dorsata). The honey bears its name after a tall Tualang tree (Koompassia excelsa) where Asian rock bees build their hives. Tualang tree can be found mostly in the tropical rain forest in the northern peninsular Malaysia, southern Thailand and Borneo. TH has been reported to contain many bioactive compounds which include benzoic acid, gallic acid, syringic acid, p-coumaric acid, trans-cinnamic acid, caffeic acid, ferulic acid, chlorogenic acid, catechin, quercetin, kaempferol, naringenin, pinobanksin-3-O-propionate and pinobanksin-3-O-butyratengenin [12, 13]. TH has also been shown to have beneficial effect at a dose of 1.0 g/kg body weight in diabetic [14] and ovariectomized [15] rats. Studies have demonstrated that TH could improve spatial learning and memory performance during chronic cerebral hypoperfusion-induced neurodegeneration [16] and induce protective effect against chronic cerebral hypoperfusion-induced neurodegeneration [17]. In addition, studies have reported that TH consumption could deter hippocampal morphological impairment in adult male rats [18] and improve hippocampal morphological impairment in ovariectomized rats [19]. In light of these data, we hypothesized that the protective effect of TH could be partly mediated through ameliorating the oxidative stress, afforded by it’s rich phenolic and flavanoid antioxidants.

Despite the growing body of evidence on TH antioxidant and anti-inflammatory actions, its neuroprotective activity in the animal model of KA-induced excitotoxicity has remained unexplored. Therefore, this study was carried out to investigate the potential neuroprotective effect and its possible mechanism of TH against the KA-induced oxidative stress and neurodegeneration on rat cerebral cortex in an animal model of KA-induced excitotoxicity. Moreover, the significance of a blockade of the cyclooxygenase (COX) pathway and the modulatory effect on Kainate receptors (KARs) were also determined by comparing the pretreatment between TH and aspirin (ASP) (non-selective inhibitor of COX) and between TH and topiramate (TPM) (its modulatory effect on KARs) to extend our understanding of the pharmacological intervention of TH. Previous studies have suggested that ASP and TPM appeared to alleviate the neurotoxicity induced by KA [20, 21].

## Methods

The experimental protocol was reviewed and approved by the Animal Ethic Committee of USM [USM/Animal Ethics Approval/2011/(68) (305)]. All procedures were conducted according to guidelines approved by the Animal Ethic Committee of USM. All efforts were made to minimize the number of animals used necessary to produce reliable scientific data and to lessen animal suffering. All the section of this report was written up in accordance with the Animal Research: Reporting in Vivo Experiment (ARRIVE) Guidelines for reporting animal research [22]. A completed ARRIVE guidelines checklist is included in Additional file 1.

## Experimental animals

Male, Sprague–Dawley rats, aged 8 weeks were obtained from Animal Research and Service Center (ARASC), Universiti Sains Malaysia (USM) Health Campus. Animals were housed individually in polypropylene cages with soft bedding in the ARASC housing facility. They were maintained in a well-ventilated animal room at temperature of 21 ± 2 °C under a standard animal room condition of a 12-h light/dark cycle. Animals had free access to drinking water and food pellets ad libitum. Animals were acclimatized for at least a week and they were observed closely for any abnormality to ensure that they were in good health before starting the experiment. No abnormalities were observed.

## Materials

All chemicals and reagents used in this study were of analytical grade. KA and ASP were purchased from Sigma-Aldrich Co., St. Louis, MO. Tualang honey (AgroMas®) was supplied by Federal Agricultural Marketing Authority (FAMA), Kedah, Malaysia. TPM was purchased from Tokyo Chemical Industry Co., Ltd., Japan. Fluoro Jade C (FJC) powder was purchased from Histo-Chem Inc., USA. Cresyl-violet powder was purchased from Merck, Germany.

### Experimental design

Male, Sprague–Dawley rats, (aged 9 – 11 weeks) weighing (mean ± SD) 290 ± 30 g were used in this study. Two set of animals were carried out; one set was for study 1; behavioral and biochemical studies (*N* = 90) and another set was for study 2; histology study (*N* = 90). For each set, animals were randomly divided into five major groups (*n* = 18/group) and each major group was further randomly divided into three subgroups depending on the time of sacrifice (after 2 h, 24 h and 48 h of KA administration), with a total of 15 subgroups (*n* = 6/subgroup). All treatments were conducted at the day session (between 8 am and 10 am) and at the night session (between 8 pm and 10 pm) for each treatment day.

i. Group 1: The saline-treated group (control group) - Animals were treated orally with drinking water five times every 12 h.
ii. Group 2: The KA only-treated group - Animals were treated orally with drinking water five times every 12 h.
iii. Group 3: The TH + KA-treated group -Animals were treated orally with TH (1.0 g/kg body weight) five times every 12 h. The TH dose of 1.0 g/kg body weight was chosen as it was previously shown to have beneficial effect in diabetic [14] and ovariectomized [15] rats.
iv. Group 4: The TPM + KA-treated group - Animals were treated orally with TPM (40 mg/kg body weight) five times every 12 h. The dosage was chosen based on other studies using topiramate [23–25].
v. Group 5: The ASP + KA-treated group - Animals were treated orally with ASP (7.5 mg/kg body weight) five times every 12 h. The dosage was chosen based on Kim et al. study [20].

### Kainic acid administration

Thirty minutes after the last administration of drinking water or honey or drugs respectively, KA (15 mg/kg body weight; 10 mg/ml in normal saline) was administered subcutaneously (s.c.) to all groups except for the control group, which received subcutaneous injection of normal saline. The dose of KA (15 mg/kg body weight; s.c.) has been previously reported to be sufficient to inflict a significant injury in the different regions of the brain (i.e. amygdala, hippocampus and cortex) and all tested parameters (i.e. citrulline level, total adenine nucleotides and total creatine compounds), to reach their peaks after 2 h following KA administration [26].

### Seizure characteristics

Following KA administration, animals were monitored for 4 h and the time to the onset of first generalized tonic-clonic seizures (characterized by stage 4 described by Zhang et al. [2]) was recorded. Since this study used single high dose KA treatments which were associated with a high mortality rate, special efforts were made to improve the survival rate of KA-treated animals. To reduce mortality [27], diazepam (10 mg/kg body weight; Atlantic Laboratories Corp. Ltd., Thailand) was administered intraperitoneally, approximately 90 min after the first generalized seizure to animals of 24 h and 48 h subgroup, except for the control groups, which received an equivalent amount of normal saline. Subcutaneous normal saline injection was administered periodically during the first 24 h recovery period.

### The open-field test procedure

For the study 1 only, behavioral change was observed using an open field test (OFT) to assess the locomotor activity of animals. With the exception of 2 h subgroups, locomotor activity of animals was documented in the 24 h and 48 h subgroups from each major group. The animals were tested one at a time and handled by their tails at all times. The animals were individually placed at the central square of the OFT apparatus (90 cm x 90 cm x 27 cm, consisting of 25 equally sized squares measuring 18 cm x 18 cm each). Animals were allowed to explore freely the area for 15 min while the locomotor activity was recorded using the video-tracking system. After each trial, the animals were returned to their cage and the apparatus was cleaned between each rat using 30% ethyl alcohol and allowed to dry between trials. All tests were conducted between 8 am and 2 pm. The OFT was performed using an in-house built-in OFT apparatus from the Department of Neurosciences, School of Medical Sciences Universiti Sains Malaysia, Kelantan, Malaysia and SMART software (Panlab, Harvard) from BRAINetwork, Universiti Sains Malaysia, Kelantan, Malaysia. Locomotor activity of animals was assessed based on the number of line crossing (the frequency of crossing one of the black grid lines with all four paws) in the OFT apparatus in 15 min duration. The behavioral of animals were observed and analyzed by an observer blinded with respect to the experimental groups.

### Preparation of tissue samples

For the study 1, depending on the time of sacrifice (after 2 h, 24 h and 48 h of KA administration), following the completion of the behavioral experiment, all major group of rats were decapitated using guillotine (Harvard Apparatus, USA) after 2 h, 24 h and 48 h of KA administration with the respective control groups. The cerebral cortex was rapidly, carefully removed, weighed and thoroughly washed with an ice-cold (0° - 4 °C) normal saline to remove any excess blood. The cerebral cortex was minced to small pieces and homogenized to make 7.5% (w/v) homogenates in ice-cold 0.1 M sodium phosphate buffer, Ph 7.4, using ice-chilled glass-homogenizing vessel in a tissue homogenizer fitted with Teflon pestle (Glas-Col, USA) at 900 rpm for 90 s. Then, the homogenates were centrifuged in a refrigerated centrifuge at 4000 rpm for 15 min at 4 °C to remove nuclei and debris. The resulting supernatant fraction of homogenates was used for the measurement of lipid peroxidation and total antioxidant status.

For the study 2, depending on the time of sacrifice (2 h, 24 h and 48 h of KA administration), all major groups of rats were deeply anesthetized with a mixture of ketamine/xylazine (90 mg/kg body weight ketamine and 5 mg/kg body weight xylazine), via intramuscular injection. The toe pinch-response method was used to determine the depth of anesthesia and animals must be unresponsive before commencing perfusion process. The anaesthetized rat was transcardially perfused with ice-cold phosphate buffer saline, pH 7.4 and followed by ice-cold 4% paraformaldehyde in 0.2 M sodium phosphate buffer, pH 7.4 after 2 h, 24 h and 48 h of KA administration with the respective control groups. Brains were rapidly removed from skulls. The cerebrum was dissected and separated from the rest of the brain and then immersed in the same fixative solution at 4 °C for overnight. After post-fixation, the cerebrum was processed, embedded in paraffin and sectioned into serial 5-μm-thick coronal sections, taken from 4.60 mm and 3.60 mm with respect to a relative distance from Bregma [28]. Prior to histochemical staining, the sections were mounted onto poly-L-lysine coated slides and were air dried for overnight. The slides were stained with cresyl-violet stain and Fluoro Jade C (FJC) to examine degenerating neurons.

### Determination of thiobarbituric acid reactive substances and total antioxidant status level

The measurement of lipid peroxidation was determined by estimating Thiobarbituric Acid Reactive Substances (TBARS), according to the method previously described by Ohkawa et al. [29] with some modifications. TBARS level (lipid peroxidation) was expressed as nanomoles of Malondialdehyde equivalent per g wet tissue (nanomoles/g wet tissue). Meanwhile, total antioxidant status (TAS) of the homogenate sample was determined as previously described by Koracevic et al. [30]. TAS level was expressed as nanomoles of uric acid equivalent per g wet tissue (nanomoles/g wet tissue).

### Evaluation of viable cells using cresyl violet staining

Cresyl violet staining was performed to provide the estimation of neuronal loss. Cresyl violet staining stock solution was prepared by dissolving 0.1 g cresyl violet acetate powder (Merck, Germany) in 75 ml of distilled water. Then, 6 ml of cresyl violet stock solution was diluted with 50 ml of 0.1 M acetate buffer (pH 3.6) to make cresyl violet working solution.

In brief, slides were treated sequentially with the following steps. First, the sections were deparaffinized with xylene and a series of descending grade of ethanol (100%, 95% and 80%) and then brought to water. Following tissue hydration, the sections were stained with cresyl violet and then rinsed with distilled water. The sections were air dried overnight, cleared in xylene and then cover slipped with Dibutylphthalate Polystyrene Xylene (DPX) mounting media (Sigma, USA).

The cresyl violet-stained sections were visualized and imaged using Olympus BX41 microscope equipped with a high resolution digital camera system and a desktop computer preinstalled with image analysis software, analySIS FIVE. Viable cells were counted and defined as cells with normal morphology, exhibiting round nuclei stained with cresyl violet-stained.

### Evaluation of degenerating neurons using Fluoro Jade C staining

In order to further evaluate neuronal degeneration in the cortex of rats, FJC histochemical staining was performed using the method described by Schmued et al. [31]. FJC is an anionic fluorescent dye, a specific marker of degenerating neurons. FJC staining stock solution was prepared by dissolving 10 mg of dry powder FJC (Histo-Chem Inc., USA) in 100 ml distilled water. Then, 10 ml of stock solution was added to 90 ml of 0.1% acetic acid, producing 0.001% concentration of FJC working solution.

In brief, slides were then treated sequentially with the following steps: sections were deparaffinized with xylene and a series of descending graded series of ethanol (100%, 95% and 80%) and then immersed in distilled water. After tissue hydration, slides were immersed in 0.1% potassium permanganate solution for 20 min with slow shaking in the dark and then rinsed with distilled water for 2 min. Slides were incubated in 0.001% FJC in 0.1% acetic acid for 10 min with slow shaking in the dark. Then, slides were rinsed 3 times with distilled water. The sections were air dried overnight, cleared in xylene and then cover slipped with DPX mounting media (Sigma, USA).

The FJC-labelled sections were visualized and imaged using an Olympus BX41 fluorescence microscope using a filter system designed for visualizing fluorescein (Excitation peak:4 nm, Emission peak: 525 nm) equipped with a high resolution digital camera system and desktop computer preinstalled with image analysis software, analySIS FIVE. Fluoro-jade-positive cell were counted and defined as a bright green fluorescence, while normal neurons appeared even darker than background with lightly staining nucleoli or were unstained.

### Neuronal quantification

Three representative sections of the cerebrum were selected for each animal that were used to quantify neuronal degeneration. These sections were taken from Bregma 4.60 mm to Bregma 3.60 mm [28] at a regular space interval of 100 μm with 5 μm thick for each rat from each group. Using analySIS FIVE image analysis software, five non-overlapping areas were identified on piriform cortex and photographed at 200x magnification. The data of these images were saved into computer. Three of these images were randomly selected for neuronal quantification. Neuronal cell counts were performed using Image J image analysis software (National Institute of Health, USA) and the data were then averaged for each animal. The neuronal quantification was performed by an observer blinded with respect to the experimental groups.

### Statistical analysis

All data were analyzed using SPSS software version 22 (SPSS Statistics IBM, Chicago, USA). Statistical significance of differences was determined by Kruskal-Wallis followed by Mann–Whitney U (MW) test. Data was considered statistically significant when p value was less than 0.05 (*p* < 0.05). Results are expressed as median (Interquartile Range; IqR).

## Results

### Administration of KA induced seizures

Administration of KA (15 mg/kg body weight, s.c.) induced epileptic seizures in all KA-treated rats and KA-treated groups that received pretreatment of TH, ASP and TPM. No seizure behavior was observed in the control group. The onset of seizure occurred most frequently within 1 h to 4 h post-KA administration. All KA-treated rats exhibited progressive motor seizures. It started with staring spells in which rats seemed to be in a motion arrest. This was followed by wet dog shakes which progressively became frequent in nature. Then, the animals displayed hyperactive behavior in which they had repeated head nodding with the intervals of continuous walking and chewing. After that, rats started to rear up on their hind limbs and then developed frequent and prolonged rearing, accompanied by forelimb clonic jerks, salivation and white foaming at the mouth (first generalized seizure). Next, the rats were falling or had loss of balance while rearing. These rearing and falling episodes continued until the rats were injected with diazepam (10 mg/kg body weight) approximately 90 min following the onset of the first generalized seizure. The rats in the control group displayed normal behavior such as walking, sniffing, grooming and exploring.

Statistical analysis indicated that there was no significant difference (*p* > 0.05) in the time to the onset of the first generalized seizures between groups, [χ2 (3) = 4.72, (*N* = 48), *p* = 0.1937] (Table 1). The median values of the time to the onset of the first generalized seizures in the KA-induced rats pre-treated with TH, ASP and TPM were no significantly different (*p* > 0.05) from those of the KA-induced rats. Therefore, pretreatment with TH, ASP and TPM did not have any apparent anticonvulsant effect in KA-induced seizures.

**Table 1:** Effect of KA-induced excitotoxicity on the time to the onset of the first generalized seizures between groups

Group	Onset time of the first generalized seizures (min) Median (IqR) (*n* = 12)	Kruskal-Wallis test (*N* = 48)	
		χ2 stat (df)	*p* value
KA	76.50 (38.50)	4.72 (3)	0.1937
TH + KA	65.00 (23.50)		
TPM + KA	64.50 (23.50)		
ASP + KA	62.50 (13.25)		

The results were expressed as the median (IqR). The significant difference was determined by non-parametric test, Kruskal-Wallis test. Mann-Whitney U test was not performed because there was no significant difference (*p* > 0.05) in the median values with Kruskal-Wallis test

### Effect of KA-induced excitotoxicity on the number of line crossing in the OFT

Statistical analysis of number of line crossing in the OFT indicated that there was a significant difference (*p* < 0.05) in the number of line crossing between the groups for all 24 h and 48 h subgroups of KA administration, as shown in Table 2 [24 h subgroups = χ2 (4) =19.79, (*N* = 30), *p* = 0.0005; 48 h subgroups = χ2 (4) = 11.71, (*N* = 30), *p* = 0.0196]. Post hoc test revealed there was a significant difference (*p* < 0.05) in the number of line crossing in the OFT between the control and KA-treated groups in 24 h and 48 h subgroups, and between the control and KA-induced groups pre-treated with TPM for 24 h subgroups of KA administration. In the OFT, KA-treated group was increased in the locomotor activity (based on number of line crossing) and hyperactivity.

**Table 2:** Effect of KA-induced excitotoxicity on the number of line crossing in the OFT

Subgroups	Number of line crossing Median (IqR) (*n* = 6)					Kruskal-Wallis test (*N* = 30)	
	CONTROL	KA	TH + KA	TPM + KA	ASP + KA	χ2 stat (df)	*p* value
24H	190.00 (57.00)	259.50 (50.50)^a^	160.50 (45.50)^b^	101.00 (57.75)^a,b^	120.00 (170.25)^b^	19.79 (4)	0.0005
48H	143.00 (41.75)	197.50 (21.25)^a^	135.00 (20.75)^b^	169.00 (153.25)	123.50 (82.00)^b^	11.71 (4)	0.0196

The results were expressed as the median (IqR). The significant difference was determined by non-parametric test, Kruskal-Wallis test followed by Mann-Whitney U post-hoc test with *p* < 0.05 indicates statistically significant difference. ^a^ *p* < 0.05 versus control group (MW); ^b^ *p* < 0.05 versus KA group (MW)

There was also a significant difference (*p* < 0.05) in the number of line crossing in the OFT between KA-treated groups and KA-induced groups pre-treated with TH of 24 h and 48 h of KA administration (Table 2). This indicated that the increased locomotor activity induced by KA was attenuated by TH pre-treatment. There was a significant difference (*p* < 0.05) in the number of line crossing in the OFT between KA-treated groups and KA-induced groups pre-treated with TPM for 24 h subgroups of KA administration, and between KA-treated groups and KA-induced groups pre-treated with ASP of 24 h and 48 h of KA administration.

### Effect of KA-induced excitotoxicity on TBARS level in the cerebral cortex

Statistical analysis of the TBARS level in the cerebral cortex indicated that there was a significant difference (*p* < 0.05) in the TBARS level between the groups for all 2 h, 24 h and 48 h subgroups of KA administration, as shown in Table 3 [2 h subgroups = χ2 (4) = 22.63, (*N* = 30), *p* = 0.0002; 24 h subgroups = χ2 (4) = 24.18, (*N* = 30), *p* = 0.0001; 48 h subgroups = χ2 (4) = 22.97, (*N* = 30), *p* = 0.0001].

**Table 3:** Effect of KA-induced excitotoxicity on TBARS level in the cerebral cortex

Subgroups	TBARS (nanomoles/g wet tissue) Median (IqR) (*n* = 6)					Kruskal-Wallis test (*N* = 30)	
	CONTROL	KA	TH + KA	TPM + KA	ASP + KA	χ2 stat (df)	*p* value
2H	11.95 (2.90)	21.93 (1.78)^a^	15.40 (3.72)^b^	15.35 (4.08)^b^	18.35 (1.31)^a,b^	22.63 (4)	0.0002
24H	13.54 (3.38)	101.55 (11.48)^a^	75.70 (8.25)^a,b^	80.19 (9.17)^a,b^	82.38 (6.69)^a,b,c^	24.18 (4)	0.0001
48H	12.55 (2.21)	81.39 (10.87)^a^	54.21 (9.32)^a,b^	57.64 (5.14)^a,b^	56.46 (8.44)^a,b^	22.97 (4)	0.0001

The results were expressed as the median (IqR). The significant difference was determined by non-parametric test, Kruskal-Wallis test followed by Mann-Whitney U post-hoc test with *p* < 0.05 indicates statistically significant difference. ^a^ *p* < 0.05 versus control group (MW); ^b^ *p* < 0.05 versus KA group (MW); ^c^ *p* < 0.05 versus KA + TH group (MW)

Post hoc test showed that there was a significant difference (*p* < 0.05) in the TBARS level between the control and all the treatment groups that received KA, for all 2 h, 24 h and 48 h subgroups of KA administration, except for between control groups and KA-induced groups pre-treated with TH and TPM after 2 h of KA administration. The analysis indicated that the TBARS level in KA- treated, TH + KA treated, TPM + KA treated and ASP + KA treated groups were significantly higher as compared with relative control groups after 2 h, 24 h and 48 h following KA administration, respectively (Table 3).

There was also a significant difference (*p* < 0.05) in the TBARS level between KA-treated groups and KA-induced groups pre-treated with TH after 2 h, 24 h and 48 h of KA administration (Table 3). This showed that TH pre-treatment significantly attenuated an increase of TBARS level induced by KA. There was also a significant difference (*p* < 0.05) in the TBARS level between KA-treated groups and KA-induced groups pre-treated with TPM and ASP after 2 h, 24 h and 48 h of KA administration (Table 3). In addition, there was a significant difference in the TBARS level between KA-induced groups pre-treated with TH and KA-induced groups pre-treated with ASP after 24 h of KA administration.

### Effect of KA-induced excitotoxicity on TAS level in the cerebral cortex

Statistical analysis of the TAS level in the cerebral cortex indicated that there was a significant difference (*p* < 0.05) in the TAS level between the groups for all 2 h, 24 h and 48 h subgroups of KA administration, as shown in Table 4 [2 h subgroups = χ2 (4) = 25.13, (*N* = 30), *p* = 0.0000; 24 h subgroups = χ2 (4) = 23.69, (*N* = 30), *p* = 0.0001; 48 h subgroups = χ2 (4) = 25.36, (*N* = 30), *p* = 0.0000].

**Table 4:** Effect of KA-induced excitotoxicity on TAS level in the cerebral cortex

Subgroups	TAS (nanomoles/g wet tissue) Median (IqR) (*n* = 6)					Kruskal-Wallis test (*N* = 30)	
	CONTROL	KA	TH + KA	TPM + KA	ASP + KA	χ2 stat (df)	*p* value
2H	789.62 (31.05)	595.83 (45.37)^a^	668.80 (17.05)^a,b^	643.11 (21.43)^a,b,c^	633.84 (27.71)^a,b,c^	25.13 (4)	0.0000
24H	798.16 (17.27)	533.40 (18.52)^a^	631.66 (29.97)^a,b^	643.32 (54.40)^a,b^	601.23 (33.85)^a,b,c^	23.69 (4)	0.0001
48H	797.59 (26.24)	578.87 (8.92)^a^	657.57 (34.49)^a,b^	664.97 (41.12)^a,b^	622.71 (20.48)^a,b,c^	25.36 (4)	0.0000

The results were expressed as the median (IqR). The significant difference was determined by non-parametric test, Kruskal-Wallis test followed by Mann-Whitney U post-hoc test with *p* < 0.05 indicates statistically significant difference. ^a^ *p* < 0.05 versus control group (MW); ^b^ *p* < 0.05 versus KA group (MW); ^c^ *p* < 0.05 versus KA + TH group (MW)

Post hoc test indicated that there was a significant difference (*p* < 0.05) in the TAS level between the control and all the treatment groups that received KA, for all 2 h, 24 h and 48 h subgroups of KA administration. The results of analysis also showed that the TAS level in KA- treated, TH + KA treated, TPM + KA treated and ASP + KA treated groups were significantly lowered as compared with relative control groups after 2 h, 24 h and 48 h following KA administration, respectively (Table 4).

There was also a significant difference (*p* < 0.05) in the TAS level between KA-treated groups and KA-induced groups pre-treated with TH after 2 h, 24 h and 48 h of KA administration (Table 4). Therefore, TH pre-treatment significantly attenuated a decrease of in TAS level induced by KA.

There was also a significant difference (*p* < 0.05) in the TAS level between KA-treated groups and KA-induced groups pre-treated with ASP and TPM, respectively, after 2 h, 24 h and 48 h of KA administration (Table 4). In addition, there was a significant difference (*p* < 0.05) in the TAS level between KA-induced groups pre-treated with TH and KA-induced groups pre-treated with ASP of 2 h, 24 h, and 48 h of KA administration and between KA-induced groups pre-treated with TH and KA-induced groups pre-treated with TPM of 2 h of KA administration (Table 4). This indicated that pretreatment with TH showed a more pronounced protective effect compared with the ASP + KA-treated treated groups.

### Effect of KA-induced excitotoxicity on the number of viable cells in the piriform cortex

**Fig. 1:**
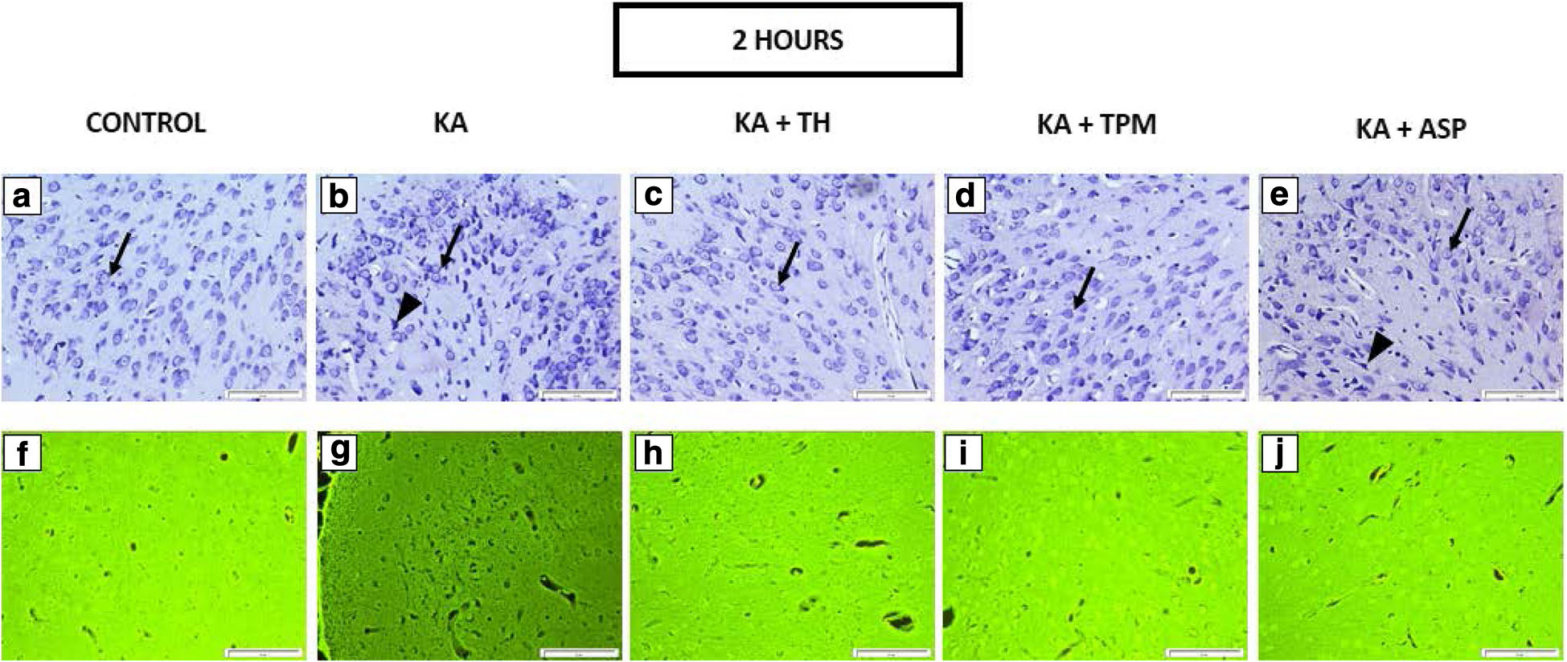
In the photomicrographs, representative image of cresyl violet staining (**a**–**e**), and FJC staining (**f**–**j**) in the piriform cortex after 2 h of KA challenge were presented. Arrows indicate the viable cell under cresyl violet staining (**a**–**e**). Viable cells were defined as cells with normal morphology, exhibiting round nuclei stained with cresyl violet-stained. In KA- treated groups and ASP+ KA treated groups, neurons appeared pyknotic, indicated by arrowheads (**b** and **e**). Neurons remained normal at 2 h after KA administration in the piriform cortex, (**f**–**j**). Scale bars: 100 μm. Magnification: 200x magnification

**Fig. 2:**
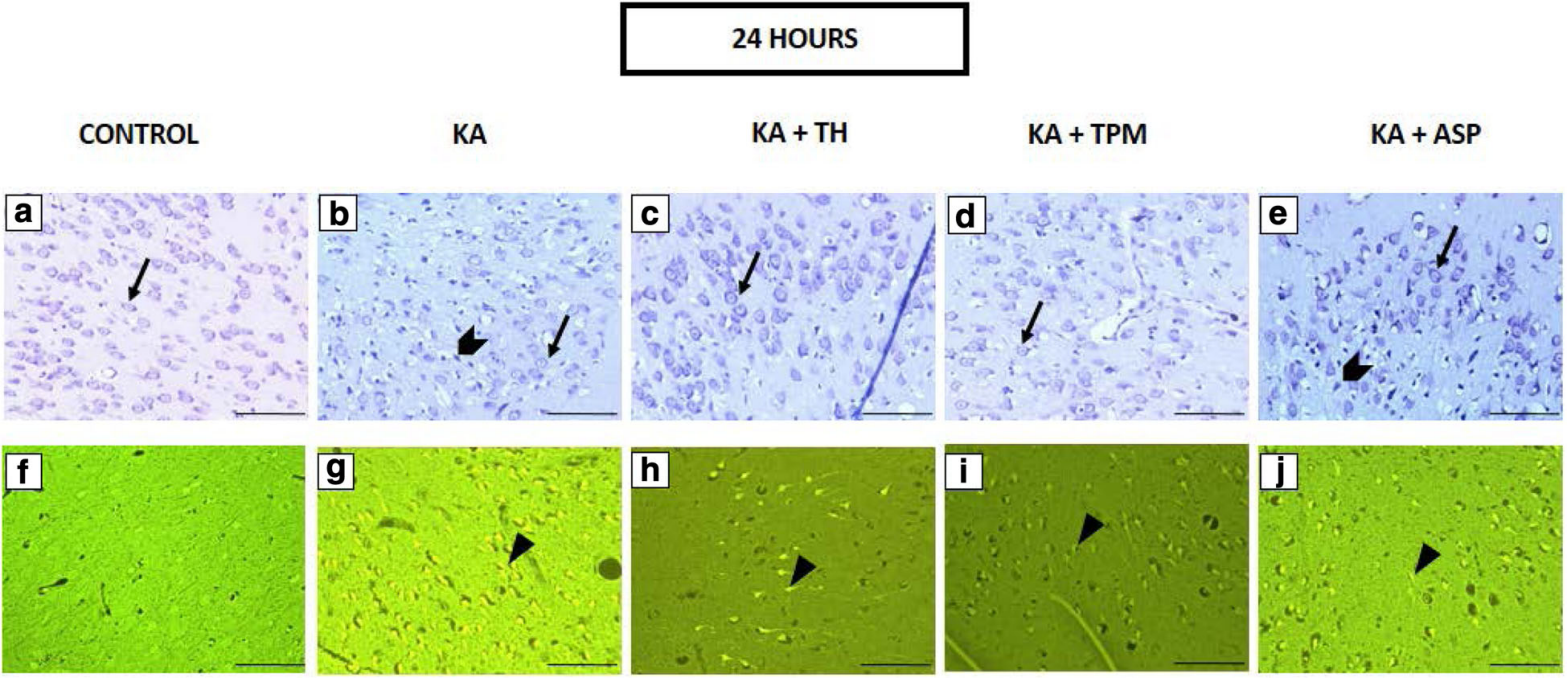
In the photomicrographs, representative image of cresyl violet staining (**a**–**e**) and FJC staining (**f**–**j**) in the piriform cortex after 24 h of KA challenge were presented. Arrows indicate the viable cell under cresyl violet staining (**a**–**e**). In KA- treated groups and ASP+ KA treated groups, neurons appeared pyknotic, indicated by chevron (**b** and **b**). Neuronal degeneration, indicated by arrowheads, was detected after 24 h of KA administration in the piriform cortex (**g**–**j**). Scale bars: 100 μm. Magnification: 200x magnification

**Fig. 3:**
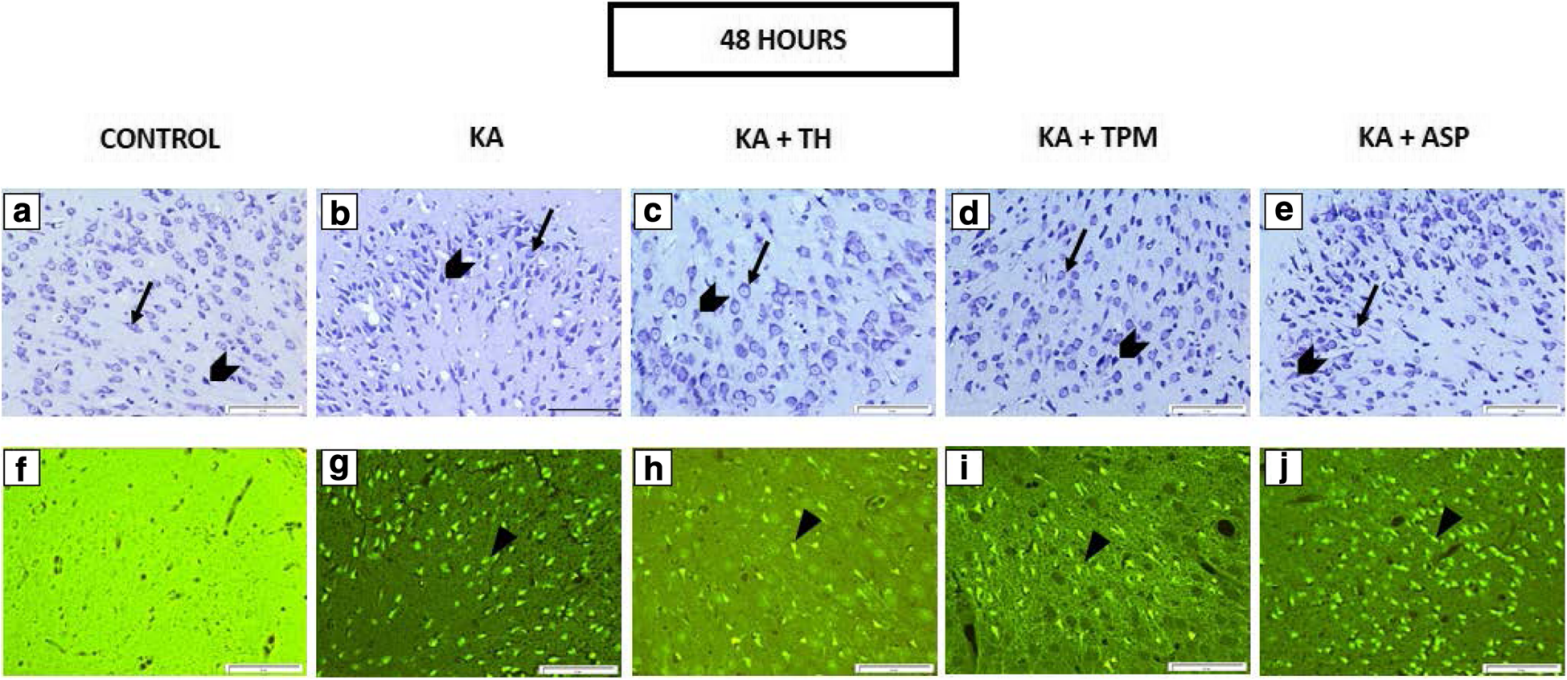
In the photomicrographs, representative image of cresyl violet staining (**a**–**e**) and FJC staining (**f**–**j**) in the piriform cortex after 48 h of KA challenge were presented. Arrows indicate the viable cell under cresyl violet staining (**a**–**e**). In KA- treated groups, TH + KA groups and TPM + KA groups, ASP+ KA treated groups, there neurons appeared pyknotic, indicated by chevron **a**–**e**). Neuronal degeneration, indicated by arrowheads, was detected after 48 h of KA administration in the piriform cortex (**f**–**j**). Scale bars: 100 μm. Magnification: 200x magnification

To evaluate the protective effect of TH, ASP and TPM against KA-induced neuronal death, we performed cresyl violet staining to quantify the number of viable cells in the piriform cortex. Systemic administration of KA caused neuronal death in the piriform cortex, as recognized by pyknotic appearance and subsequently has less viable cells, in comparison with the control groups, which have more viable cells in the piriform cortex, as shown in Fig. 1(a) -(e), Fig. 2(a) - (e), and Fig. 3(a) -(e). Viable cells were defined as cells with normal morphology, exhibiting round nuclei stained with cresyl violet.

Statistical analysis of the number of viable cells in the piriform cortex indicated that there was a a significant difference (*p* < 0.05) in the number of viable cells Table 5 [2 h subgroups = χ2 (4) = 24.46, (*N* = 30), *p* = 0.0001; 24 h subgroups = χ2 (4) = 25.96, (*N* = 30), *p* = 0.0000; 48 h subgroups = χ2 (4) = 24.58, (*N* = 30), *p* = 0.0001]. Post hoc test revealed that there was a significant difference (*p* < 0.05) in the number of viable cells between the control and all the treatment groups that received KA, for all 2 h, 24 h and 48 h subgroups of KA administration (Table 5). This indicated that there was less viable cells in KA- treated, TH + KA treated, TPM + KA treated and ASP + KA treated groups and they were significantly reduced as compared with the relative control groups after 2 h, 24 h and 48 h following KA administration, respectively (Table 5).

**Table 5:** Effect of KA-induced excitotoxicity on the number of viable cells in the piriform cortex

Subgroups	Viable cells Median (IqR) (n = 6)					Kruskal-Wallis test (*N* = 30)	
	CONTROL	KA	TH + KA	TPM + KA	ASP + KA	χ2 stat (df)	*p* value
2H	279.17 (25.33)	135.17 (53.75)^a^	202.00 (38.42)^a,b^	158.00 (3.41)^a,c^	133.00 (27.75)^a,c^	24.46 (4)	0.0001
24H	271.83 (22.24)	110.50 (31.25)^a^	153.83 (57.00)^a,b^	129.00 (9.00)^a,c^	90.34 (24.25)^a,b,c^	25.96 (4)	0.0000
48H	251.00 (40.50)	127.67 (54.42)^a^	160.84 (23.42)^a^	141.00 (14.41)^a,c^	80.00 (10.17)^a,b,c^	24.58 (4)	0.0001

The results were expressed as the median (IqR). The significant difference was determined by non-parametric test, Kruskal-Wallis test followed by Mann-Whitney U post-hoc test with *p* < 0.05 indicates statistically significant difference. ^a^ *p* < 0.05 versus control group (MW); ^b^ *p* < 0.05 versus KA group (MW); ^c^ *p* < 0.05 versus KA + TH group (MW)

There was also a significant difference (*p* < 0.05) in the number of viable cells between KA-treated groups and KA-induced groups pre-treated with TH after 2 h and 24 h of KA administration, except between KA-treated groups and KA-induced groups pre-treated with TH after 48 h of KA administration (Table 5). Therefore, TH pre-treatment significantly attenuated a decrease in the number of viable cells induced by KA.

In addition, there was a significant difference (*p* < 0.05) in the number of viable cells between KA-induced groups pre-treated with TH and KA-induced groups pre-treated with ASP, and between KA-induced groups pre-treated with TH and KA-induced groups pre-treated with TPM after 2 h, 24 h and 48 h of KA administration (Table 5). This indicated that pretreatment with TH showed a more protective effect than ASP + KA-treated and TPM + KA-treated groups.

### Effect of KA-induced excitotoxicity on the number of FJC-positive cells in the piriform cortex

In the present study, the degenerated neurons were labelled using FJC staining. Histological observation of the FJC-stained slides revealed that FJC-positive cells were absent in all control groups but were identified in all treatments groups that received KA, except in 2 h treated group, as shown in Fig. 1(f) -(j). The FJC-stained neurons, which emitted bright green fluorescence, could be identified clearly in the 24 h and 48 h post-KA administration, as shown in Fig. 2(f) - (j) and Fig. 3(f) -(j).

Statistical analysis of the number of number of FJC-positive cells in the piriform cortex indicated that there was a significant difference (*p* < 0.05) in the number of FJC-positive cells between the groups, for both of 24 h [χ2 (4) = 26.17, (*N* = 30), *p* = 0.0000] and 48 h [χ2 (4) = 24.71, (*N* = 30), *p* = 0.0001] of KA administration (Table 6). As predicted, KA administration inflicted neuronal degeneration in the piriform cortex of KA-treated groups at 24 h and 48 h after KA administration, which was not the case in the control, where there was no FJC -positive cells observed [see Fig. 1(f) -(j), Fig. 2(f) - (j) and Fig. 3(f) -(j)].

**Table 6:** Effect of KA-induced excitotoxicity on the number of FJC-positive cells in the piriform cortex

Subgroups	FJC-positive cells Median (IqR) (*n* = 6)					Kruskal-Wallis test (*N* = 30)	
	CONTROL	KA	TH + KA	TPM + KA	ASP + KA	χ2 stat (df)	*p* value
24H	0	225.50 (54.25)^a^	101.67 (30.08)^a,b^	151.33 (55.34)^a,b,c^	161.50 (53.92)^a,b,c^	26.17 (4)	0.0000
48H	0	168.67 (18.00)^a^	94.00 (7.16)^a,b^	136.67 (40.50)^a,b,c^	168.84 (32.66)^a,c^	24.71 (4)	0.0001

The results were expressed as the median (IqR). The significant difference was determined by non-parametric test, Kruskal-Wallis test followed by Mann-Whitney U post-hoc test with *p* < 0.05 indicates statistically significant difference. ^a^ *p* < 0.05 versus control group (MW); ^b^ *p* < 0.05 versus KA group (MW); ^c^ *p* < 0.05 versus KA + TH group (MW)

Further examination of the data revealed that there were significant differences (*p* < 0.05) in the number of FJC-positive cells between KA- treated groups and KA-treated groups that received pretreatment of TH, ASP and TPM at 24 h of KA administration (Table 6). The number of FJC-positive cells in the piriform cortex of 24 h of KA administration were reduced by pretreatment with TH, TPM and ASP. This indicated that pretreatment with TH, ASP and TPM did have some protective effect in KA-induced neuronal degeneration in the piriform cortex after 24 h KA administration.

At 48 h of KA administration, the quantitative analysis of FJC-positive cells showed that there was a significant difference (*p* < 0.05) between KA-treated groups and KA-induced groups pre-treated with TH and TPM but there was no significance difference (*p* > 0.05) between KA-treated and KA-induced groups pre-treated with ASP (Table 6). This suggested that TH and TPM pretreatment ameliorated the KA-induced neuronal degeneration in the piriform cortex of 48 h of KA administration but not the case in the pretreatment with ASP.

In addition, quantitative analysis of neuronal degeneration using FJC staining revealed there was a significant difference (*p* < 0.05) between KA-induced groups pre-treated with TH and KA-induced groups pre-treated with ASP and between KA-induced groups pre-treated with TH and KA-induced groups pre-treated with TPM of 24 h and 48 h of KA administration. This indicated that pretreatment with TH showed more protective effect than ASP + KA-treated and TPM + KA-treated groups.

## Discussion

In this experiment, several parameters such as behavioral changes, oxidative stress related markers, and neuronal degeneration were examined to confirm the protective effect of TH on KA-induced excitotoxicity in a rat model. The administration of KA has been shown to induce a sequence of well-characterized seizures syndrome [2, 7]. Overactivation of glutamate receptors by KA has resulted in the increased production of ROS, which are mediators of oxidative stress. Oxidative stress has been suggested to play an important role in the mechanism of excitotoxicity and neurodegeneration on the different brain regions. Oxidative stress can cause cellular damage and production of ROS, which oxidizes membrane lipids, protein and DNA. There are growing evidences suggesting that oxidative stress has been implicated in the mechanism of excitotoxicity and neurodegeneration on different brain regions after the induction of KA on rodents [5, 6]. Brain is considered to be very vulnerable to oxidative stress because of its great consumption of energy, oxygen and glucose, large amount of peroxidizable polyunsaturated fatty acids and relatively low antioxidant capability.

In the present study, we have demonstrated that administration of KA (15 mg/kg body weight; s.c.) induced epileptic seizures. All KA- treated groups and KA-treated groups that received pretreatment of TH, ASP and TPM had a status epilepticus. In the OFT, KA-treated group has an increased in the locomotor activity (based on number of line crossing) and hyperactivity. Next, we examined TBARS levels and TAS levels in the cerebral cortex after 2 h, 24 h and 48 h of KA administration. These parameters were associated with oxidative neuronal damage. TBARS levels were elevated whereas, TAS levels was decreased in KA-treated groups after 2 h, 24 h and 48 h of KA administration, as compared to control group. In addition, KA-induced epileptic seizure caused neuronal degeneration in the piriform cortex. Our result showed that degenerated neurons were not detected after 2 h but after 24 h and 48 h of KA administration in the piriform cortex.

Previous reports have shown that lipid peroxidation was enhanced by KA in the piriform cortex after 8 h and 16 h, continued to increase and gradually decreased after 48 h and 5 days [6, 21, 32]. The elevated TBARS levels after 2 h of KA administration suggest an increased lipid peroxidation during an early phase of the excitotoxic damage. Sarkar et al. [33], Riba-Bosch and Pérez-Clausell [34] and Hopkins et al. [35] have reported that degenerating neurons in the piriform cortex could be detected after 4 h, 6 h and 8 h of KA administration and degenerating neurons continued to increase in number throughout the affected areas, reaching its peak at 2 weeks and gradually decreased by 2 months of KA administration. This indicated that neurons in the piriform cortex were undergoing degeneration as early as 4 h of KA administration and there was a temporal progression of KA-induced neurodegeneration as its continue to increase to its peak and gradually decrease in the number as time goes by after KA administration. Since an increase in TBARS level and a decrease in TAS level occurred as early as after 2 h of KA administration, this study suggests that lipid is exposed to oxidative damage and strongly indicates the oxidative stress as a possible mechanism of neurodegeneration. Our results demonstrated that oxidative stress was enhanced by KA in the cerebral cortex after 2 h, 24 h and 48 h of KA administration.

In this study, we have demonstrated that pretreatment of TH (at dose of 1.0 g/kg body weight) could reduce KA-induced neuronal degeneration in the piriform cortex but had failed to prevent seizures induced by KA. In addition, the increased locomotor activity in the OFT was attenuated by TH pre-treatment. TH pre-treatment also significantly reduced the oxidative stress, as shown by an increase in TBARS level and a decrease in TAS level. TH has been reported to contain many bioactive compounds. Several studies have been conducted to investigate the protective effects of specific bioactive compound contained in TH against KA model of excitotoxicity. For example, gallic acid, which is one of the bioactive compound found in TH, has been shown to attenuate the severity of seizure behaviors, reduced neuronal apoptosis and reduced oxidative stress in KA-induced excitotoxicity model [36]. Another bioactive compound is caffeic acid. It has also been shown to provide protection on memory impairment and on oxidative stress induced by KA [37]. These indicate that several important compounds found in TH may play protective roles against KA-induced neuronal degeneration, which may act through their antioxidant activity. Other study on propolis, which is another beehive product, appeared to be effective in preventing KA-induced neuronal loss in the regions of the hippocampus [11]. In addition, propolis was found to attenuate KA-induced seizures and KA-induced oxidative stress in the brain thought to be due to its antioxidant property [11, 38]. These studies suggested that these antioxidants provide protection against KA-induced neuronal degeneration and seizures through antioxidant mechanism. Taken together, the beneficial of TH may be due to its antioxidant property which is attributed to the presence of flavonoids and phenolic compounds [12, 13].

This study also determined the significance of a blockade of the COX pathway and the modulatory effect on KARs, by comparing pretreatment between TH and ASP and between TH and TPM in order to extend our understanding of the pharmacological mechanism of TH. Pretreatment with TH helped to reduce the KA-induced behavioral change, oxidative stress and neuronal degeneration and it showed a more protective effect than ASP + KA-treated and TPM + KA-treated groups. Other study demonstrated that ASP alone did not have any effect on the KA-induced neurodegeneration, while combination of ASP and esculetin, an inhibitor of lipoxygenase (LOX), prevented KA-induced neuronal death [20]. In addition, the findings from Baran et al. [39] and Minutoli et al. [40] studies have suggested that the protection against KA-induced neurodegeneration via combination of COX and LOX inhibitor pathways or via dual inhibitor of COX and LOX pathways were more effective than using only either a COX inhibitor or a LOX inhibitor alone. Administration of KA has caused the activation of phospholipase A followed by an increase level of arachidonic acid [41]. Arachidonic acid then undergoes degradation by COX or LOX, which form prostaglandins and leukotriene, respectively. The free radicals are formed from prostaglandins synthesis and studies have demonstrated the formation of free radicals due to the action of KA [6, 42]. With the dual inhibition of COX/LOX, the effect of KA-induced excitotoxicity could be attenuated, which blocked both leukotriene and prostaglandins production. This augments its synergistic protective effect, directly through the anti-inflammatory mechanisms and indirectly through the anti-radical activity. This may well indicate that blocking of both COX and LOX pathways are necessary to protect the brain against KA-induced seizures and KA-induced neurotoxicity. Furthermore, studies have demonstrated that topiramate has some protective effect against KA-induced seizures and KA-induced hippocampal neurotoxicity [21, 43]. The discrepancy between the present and previous studies might probably due to differences in experimental conditions such as administration route of KA, administration route of drugs, animal age (young, middle-aged or aged), animal strain and animal species (rat or mice).

## Conclusion

Administration of KA (at a dose of 15 mg/kg body weight; s.c.) induced seizures and resulted in an increase in locomotor activity in KA-treated animals. An increased lipid peroxidation and a decreased total antioxidant status suggestive of an oxidative stress were observed during an early phase of the excitotoxic damage that preceded neurodegeneration. This study suggests that oxidative stress could be a possible mechanism of neurodegeneration and TH attenuates KA-induced oxidative stress. Hence, TH reduces neurodegeneration in the rat cerebral cortex after 2 h, 24 h and 48 h of KA administration due to its antioxidant property.

## Additional file

Additional file 1: The ARRIVE Guidelines Checklist. (DOCX 661 kb)

## Abbreviations

ASP: Aspirin
COX: Cyclooxygenase
FJC: Fluoro Jade C
IqR: Interquartile range
KA: Kainic acid
LOX: Lipoxygenase
OFT: Open field test
ROS: Reactive oxygen species
s.c.: Subcutaneous injection
TAS: Total antioxidant status
TBARS: Thiobarbituric acid reactive substances
TH: Tualang honey
TPM: Topiramate
