# Supplementary material for: Effect of tualang honey against KA-induced oxidative stress and neurodegeneration in the cortex of rats
Source: BMC Complement Altern Med. 2017 Jan 9;17:31. doi: 10.1186/s12906-016-1534-x (PMC5223557; doi:10.1186/s12906-016-1534-x)

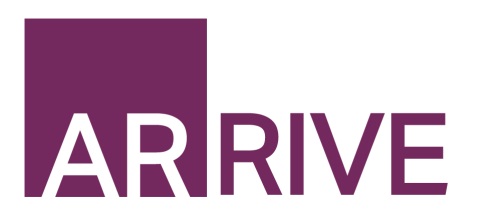


The ARRIVE Guidelines Checklist

Animal Research: Reporting In Vivo Experiments

Carol Kilkenny^1^, William J Browne^2^, Innes C Cuthill^3^, Michael Emerson^4^ and Douglas G Altman^5^

*^1^The National Centre for the Replacement, Refinement and Reduction of Animals in Research, London, UK, ^2^School of Veterinary Science, University of Bristol, Bristol, UK, ^3^School of Biological Sciences, University of Bristol, Bristol, UK, ^4^National Heart and Lung Institute, Imperial College London, UK, ^5^Centre for Statistics in Medicine, University of Oxford, Oxford, UK.*

|  | | ITEM | RECOMMENDATION | Section/ Paragraph |
| --- | --- | --- | --- | --- |
| 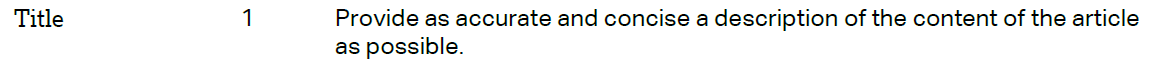 | | | 1. **Title** |  |
| 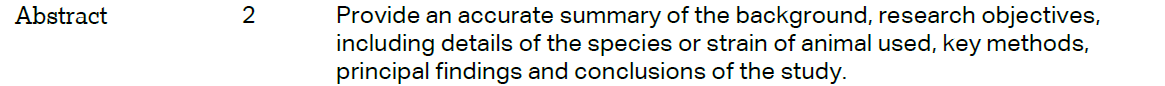 | | | 1. **Abstract** |  |
| INTRODUCTION | | |  |  |
| 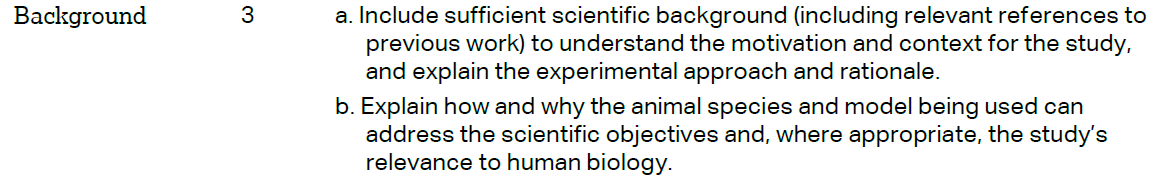 | | | - 1. **Background Section, Paragraph 1, 2,3**   2. **Background Section, Paragraph 1, 2,3** |  |
| 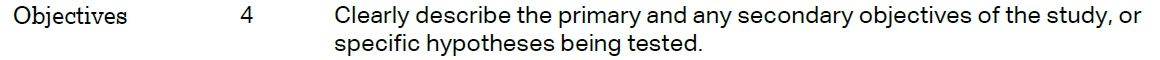 | | | 1. **Background Section, Paragraph 3** |  |
| METHODS | | |  |  |
| 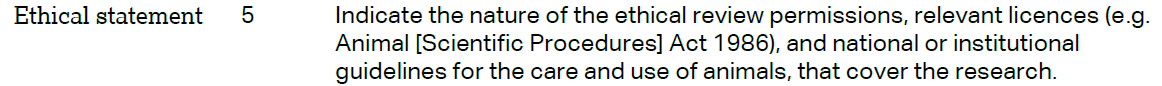 | | | 1. **Methods Section, Paragraph 1** |  |
| 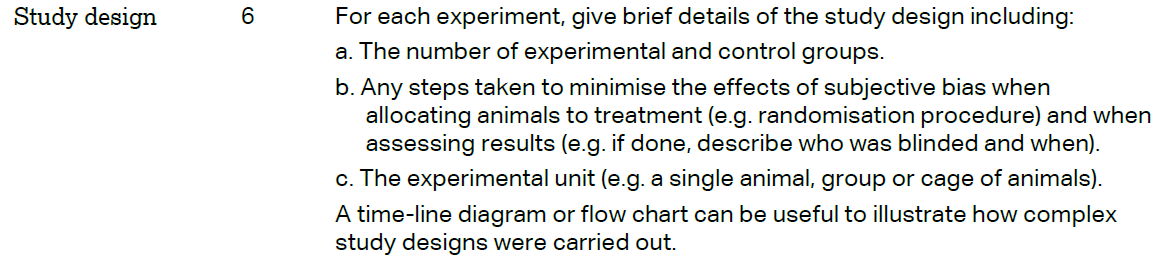 | | | - 1. **Methods Section, Experimental design subsection**   2. **Methods Section** - **Experimental design subsection** - **Seizure characteristics subsection** - **The open-field test procedure subsection** - **Neuronal quantification subsection**   1. **Methods Section** - **Experimental design subsection** - **Kainic acid administration subsection** |  |
| 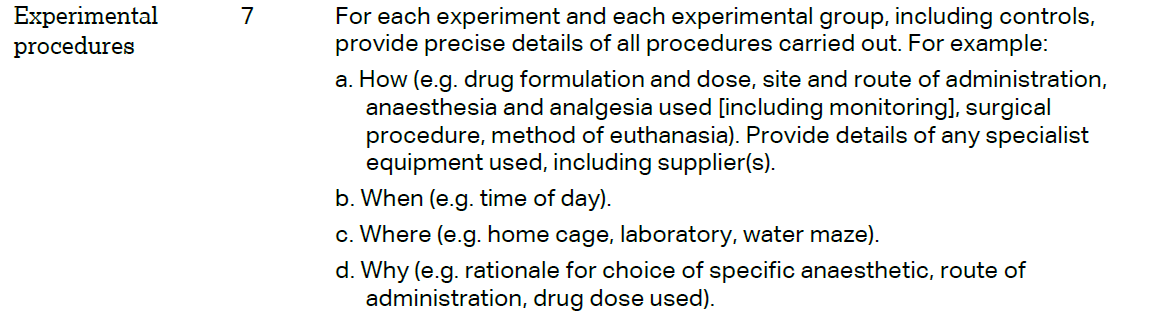 | | | - 1. **Methods Section** - **Experimental design subsection** - **Kainic acid administration subsection** - **Seizure characteristics subsection** - **Preparation of tissue samples subsection**    1. **Methods Section** - **Experimental design subsection** - **Kainic acid administration subsection** - **Seizure characteristics subsection** - **The open-field test procedure subsection**   1. **Methods Section** - **Experimental animals subsection** - **The open-field test procedure subsection**   1. **Methods Section** - **Experimental design subsection** - **Kainic acid administration subsection** |  |
| 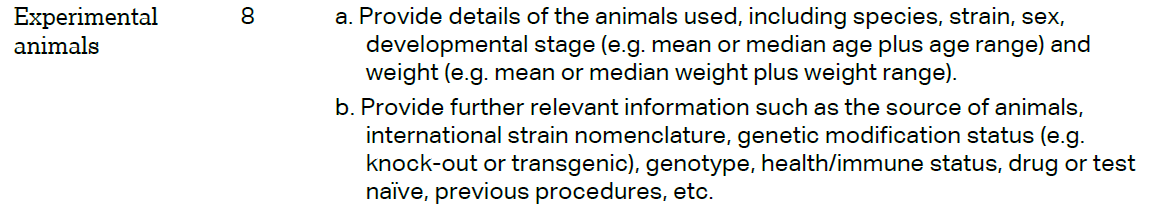 | | | - 1. **Methods Section, Paragraph** - **Experimental animal subsection** - **Experimental design subsection**   1. **Methods Section, Paragraph**   **• Experimental animal subsection** |  |

| 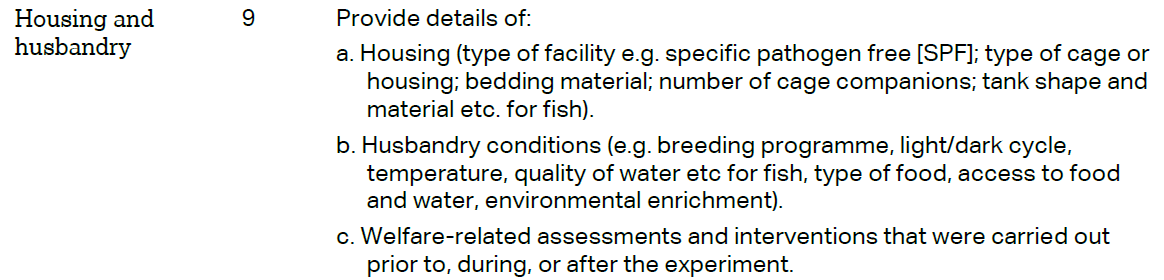 | 1. **Methods Section, Experimental animal subsection** 2. **Methods Section**  - **Experimental animal subsection**  1. **Methods Section, Paragraph**   **• Experimental animal subsection**  **• Seizure characteristics subsection** |
| --- | --- |
| 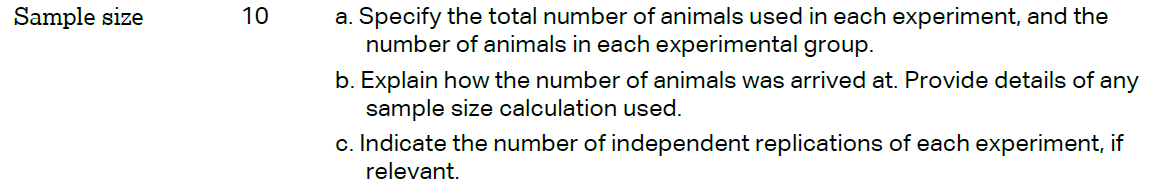 | - 1. **Methods Section, Experimental design subsection**   2. **Methods Section, Experimental design subsection**   3. **Methods Section** - **Experimental design subsection** - **Neuronal quantification subsection** |
| 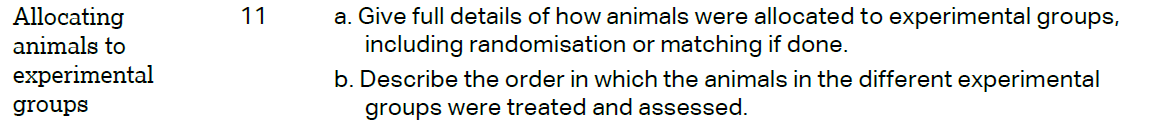 | - 1. **Methods Section, Experimental design subsection**   2. **Methods Section, Experimental design subsection** |
| 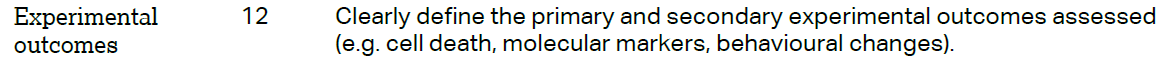 | 1. **Methods Section, Paragraph**   **• Experimental design subsection**   - **Seizure characteristics subsection** - **The open-field test procedure subsection** - **Determination of thiobarbituric acid reactive substances and total antioxidant status level subsection** - **Evaluation of viable cells using cresyl violet staining subsection** - **Evaluation of degenerating Neurons Using Fluoro Jade C staining subsection** |
| 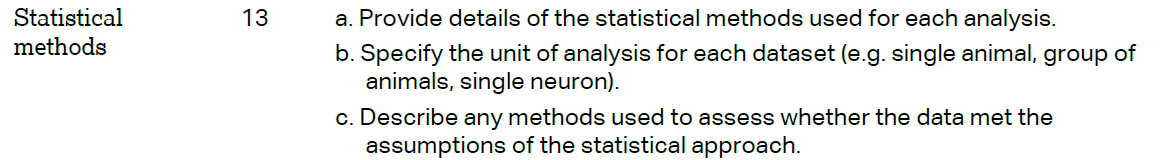 | 1. **a,b,c**    - **Methods Section, Neuronal quantification**    - **Methods Section, Statistical analysis**    - **Methods Section, Statistical analysis** |
| RESULTS |  |
| 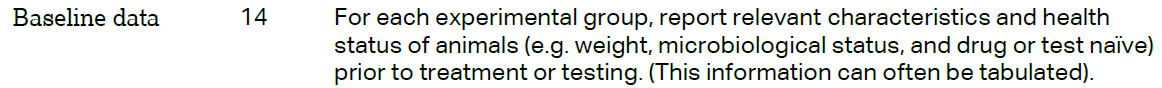 | 1. **Results Section, Administration of KA induced seizures subsection** |
| 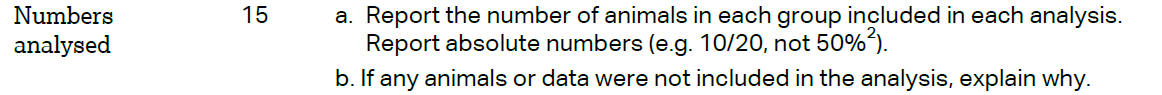 | - 1. **Throughout results Section**   2. **Not applicable** |
| 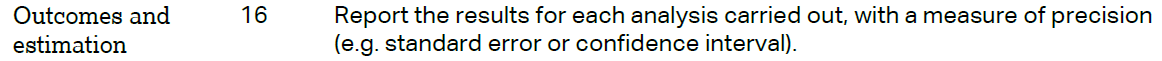 | 1. **Throughout results Section** |
| 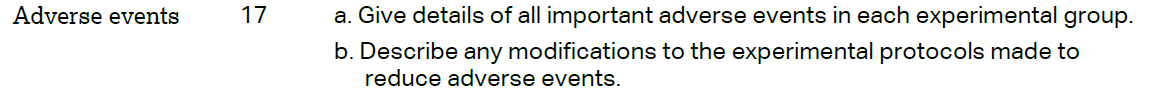 | **Not applicable** |
| DISCUSSION |  |
| 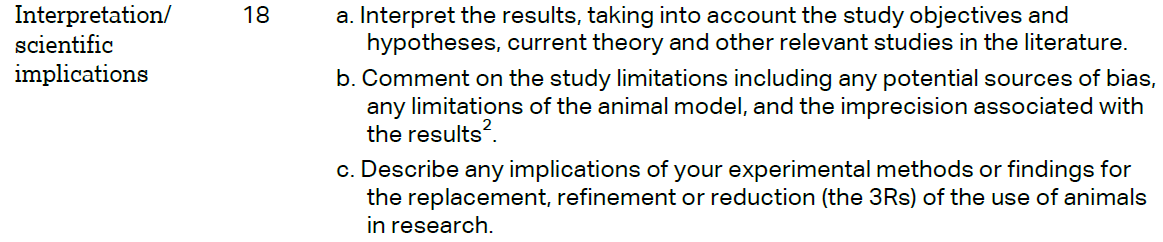 | **Throughout Discussion Section** |
| 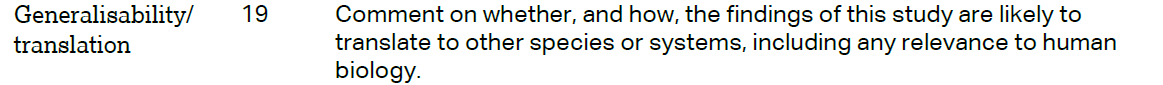 | 1. **Not applicable** |
| 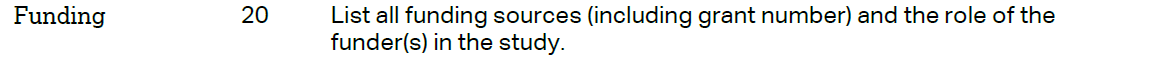 | 1. **Funding Section** |

The ARRIVE guidelines. Originally published in *PLoS Biology*, June 2010^1^
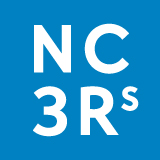

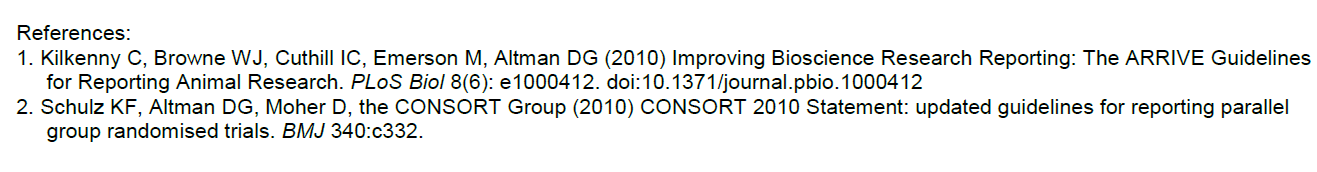

Supplement: Additional file 1: — The ARRIVE Guidelines Checklist. (DOCX 661 kb) [file 12906_2016_1534_MOESM1_ESM.docx]
